# Adjunctive Hemoadsorption Therapy with CytoSorb in Patients with Septic/Vasoplegic Shock: A Best Practice Consensus Statement

**DOI:** 10.3390/jcm12237199

**Published:** 2023-11-21

**Authors:** Steffen Mitzner, Klaus Kogelmann, Can Ince, Zsolt Molnár, Ricard Ferrer, Axel Nierhaus

**Affiliations:** 1 Division for Tropical Medicine, Infectious Diseases and Nephrology, Department of Internal Medicine, University of Rostock, 18051 Rostock, Germany; steffen.mitzner@med.uni-rostock.de; 2 Department of Anesthesiology and Intensive Care Medicine, Klinikum Leer, 26789 Leer, Germany; klaus.kogelmann@online.de; 3 Department of Intensive Care, Erasmus MC, University Medical Centre Rotterdam, 3015 GD Rotterdam, The Netherlands; c.ince@erasmusmc.nl; 4 Department of Anesthesiology and Intensive Care Medicine, Semmelweis University, 1085 Budapest, Hungary; zsoltmolna@gmail.com; 5 CytoSorbents Europe GmbH, 12587 Berlin, Germany; 6 SODIR Research Group, Intensive Care Department, Vall d’Hebron University Hospital, Vall d’Hebron Institut de Recerca, 08035 Barcelona, Spain; ricard.ferrer@vallhebron.cat; 7 Department of Intensive Care Medicine, University Medical Center Hamburg-Eppendorf, 20251 Hamburg, Germany

**Keywords:** shock, vasoplegic shock, septic shock, hemoadsorption, CytoSorb therapy, hyperinflammation

## Abstract

A dysregulated host response is a common feature in critically ill patients due to both infectious and non-infectious origins that can lead to life-threatening organ dysfunction, which is still the primary cause of death in intensive care units worldwide. In its course, pathologic, unregulated levels of inflammatory mediators are often released into the circulation, a phenomenon also referred to as a “cytokine storm”. To date, there are no approved therapies to modulate the excessive immune response and limit hyperinflammation with the goal of preventing related organ failure and death. In this context, extracorporeal blood purification therapies aiming at the alteration of the host inflammatory response through broad-spectrum, non-selective removal of inflammatory mediators have come into focus. A novel hemoadsorption device (CytoSorb^®^, CytoSorbents Inc., Princeton, NJ, USA) has shown promising results in patients with hyperinflammation from various origins. Although a significant body of literature exists, there is ongoing research to address many important remaining questions, including the optimal selection of patient groups who might benefit the most, optimal timing for therapy initiation, optimal schedule for adsorber exchanges and therapy duration, as well as an investigation into the potential removal of concomitant antibiotics and other medications. In this review, we discuss the existing evidence and provide a consensus-based best practice guidance for CytoSorb^®^ hemoadsorption therapy in patients with vasoplegic shock.

## 1. Background

A dysregulated host response is a common feature in critically ill patients due to both infectious and non-infectious origins that can lead to life-threatening organ dysfunction [1]. Despite advances in critical care, this overwhelming response of the host immune system still represents a major challenge in everyday practice, and especially within the domain of sepsis, it remains a global health and economic problem [2].

The pathophysiology of hyperinflammation-related critical illness is multifaceted and involves a complex interplay between cellular and biochemical interactions, ultimately resulting in the disruption of the well-balanced immunological state between the pro-inflammatory and anti-inflammatory forces. It is mediated by the activation of the innate immune system and typically results in a hyperinflammatory state, which is characterized by an overwhelming production and release of inflammatory mediators that are often referred to as a “cytokine storm” [3]. The binding of pathogen or damage-associated molecular patterns (PAMPs and DAMPs, respectively) to specific receptors induces a complex intracellular signaling system with the activation of pro- and anti-inflammatory pathways [4], leading to endothelial barrier dysfunction, disturbances of microcirculation, vasodilatation, progressive tissue damage, and multiple organ dysfunction [5]. The most extensively studied dysregulated inflammatory condition leading to critical illness is, without doubt, sepsis and septic shock.

**Figure 1:**
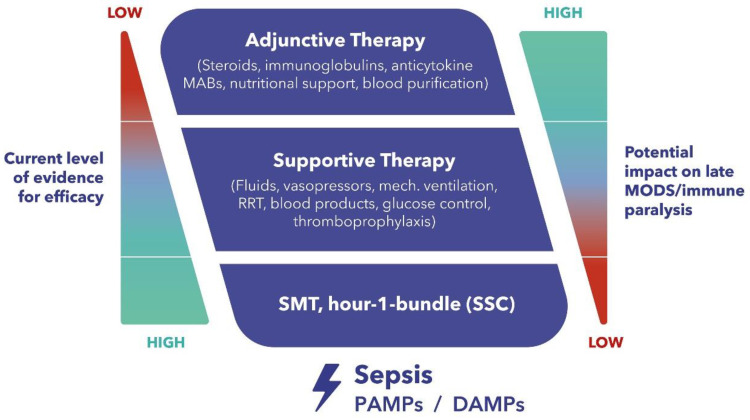
Therapeutic Concepts in Sepsis and Septic Shock—Dilemma and Challenge. MABs, monoclonal antibodies. RRT, renal replacement therapy. MODS, multiple organ dysfunction syndrome. SMT, standard medical therapy. SSC, surviving sepsis campaign. PAMPs, pathogen-associated molecular patterns. DAMPs, damage-associated molecular patterns.

The current standard therapeutic approach during the early stage of septic shock is well defined and is also supported by substantial evidence [6]. However, when standard medical therapy and advanced organ support fail to improve the patient’s condition after several hours of guideline-directed treatment, adjunctive therapies, which are aimed at modulating the host response, may be considered (Figure 1). However, despite considerable advances in its management, most sepsis trials investigating promising technologies and drugs that modulate the inflammatory response have failed in the past to produce convincing results [4]. One of the potential adjunctive therapies is extracorporeal hemoadsorption, of which the most studied platform is CytoSorb^®^ [7]. Notwithstanding considerable data that has been generated over the years, definitive evidence is still lacking, despite the increasing number of treatments worldwide. Furthermore, especially in early studies, management of CytoSorb^®^ therapy was not always performed in an optimal manner, which might have confounded the observed results. Ongoing international registries like COSMOS (CytoSorb Treatment of Critically Ill Patients) are extremely important for collecting prospectively real-world clinical use patterns of the CytoSorb^®^ device. Therefore, our aim is to elucidate the potential role and best clinical practice of immunomodulation by extracorporeal inflammatory mediator removal with CytoSorb^®^ in this patient population.

## 2. Potential Role of Extracorporeal Cytokine Adsorption

Although extracorporeal cytokine adsorption as an adjunctive therapy has been introduced predominantly for sepsis [8,9,10], the therapeutic potential of hemoadsorption likely transcends sepsis, since hyperinflammation is common to many other pathologies. In addition to bacterial infections, viral [11,12], fungal [13,14], and protozoan [15], infections can also result in a dysregulated host response and systemic hyperinflammation.

As demonstrated in a large cohort of 1886 hospitalized patients with community-acquired pneumonia (CAP), those with concurrently high levels of both pro- and anti-inflammatory cytokines have the highest risk of death [16]. These findings suggest that excessive production of cytokines plays a crucial, and potentially harmful role in the process. Therefore, removing inflammatory mediators from the circulation in a balanced, concentration-dependent manner has a pathophysiological basis; restoring the physiological immune response may foster improved organ function(s) and expedite recovery. Additionally, non-infectious conditions with systemic inflammatory repercussions, such as major burns [17,18], major trauma [19], severe pancreatitis [20], and major surgery [21,22] can exhibit many of the clinical features of hyperinflammation. Hemoadsorption has proven notably effective in treating patients with crush syndrome who have rhabdomyolysis, with CytoSorb^®^ successfully eliminating both myoglobin and creatine kinase [23]. In an interesting case study by Dilken and coworkers, the importance of timing was demonstrated where the late application of CytoSorb^®^, while being highly effective in removing myoglobin, was too late to reverse the clinical deterioration of the patient [24].

## 3. The CytoSorb Adsorber

### 3.1. Properties of the Device

Properties of the CytoSorb^®^ technology are based on highly biocompatible, porous polymer beads designed to capture and adsorb primarily hydrophobic substances in the middle molecular range with a size selectivity up to approximately 60 kDa, a range where most cytokines reside. CytoSorb^®^ hemoadsorption beads are polystyrene-divinylbenzene porous particles (average particle diameter 450 μm, 0.8–5 nm pore diameter, 850 m^2^/g surface area) with a biocompatible polyvinyl-pyrrolidone coating [25]. These beads create a tightly structured network within the cartridge, and the surface relevant for adsorption is located inside the beads. The substances must be small enough to enter the beads and hydrophobic to form the necessary physico-chemical interactions with the polymer. Removal is concentration-dependent (“autoregulation”), indicating that high removal efficiency takes place only at elevated concentrations [26]. The use of the device is not associated with significant removal of albumin, coagulation factors, and immunoglobulins [27], there is also no activation of the coagulation and complement system and only minor and transient reduction in platelets. Overall, the device has a favorable safety profile with more than 200,000 treatments delivered worldwide to date across a wide range of critical care conditions [28].

### 3.2. Effects of CytoSorb Therapy on Circulating Cytokines

Over the past several years, extensive *in vitro* modeling and *in vivo* testing have been performed on the CytoSorb^®^ polymer, miniaturized CytoSorb^®^ cartridges and the commercially available CytoSorb^®^ 300 mL device, to determine its ability to remove both endogenous (e.g., cytokines) as well as exogenous compounds (e.g., drugs).

As shown in a multitude of studies, CytoSorb^®^ is able to effectively reduce serum levels of pro- and anti-inflammatory mediators as well as other molecules involved in the inflammatory process (tumor necrosis factor -TNF-α, interleukin -IL-1β, IL-6, IL-10, NF-κB, chemokines CXCL-1, Mb and CCL2) in both animal [29,30,31,32] and human studies [33]. In the recent study by Jansen et al., clear proof of the mechanistic efficacy of cytokine removal was demonstrated in healthy volunteers who were administered endotoxin. Compared to the control group, CytoSorb^®^ significantly lowered the plasma levels of TNF-α (−58%, *p* < 0.0001), IL-6 (−71%, *p* = 0.003), IL-8 (−48%, *p* = 0.02), and IL-10 (−26%, *p* = 0.03) after an endotoxin challenge. Furthermore, no negative long-term consequences on immunocompetence by the intervention were observed a week later after the second endotoxin challenge [33].

### 3.3. Effects of CytoSorb Therapy on Clinical Parameters

One of the most consistent effects of CytoSorb^®^ hemoadsorption therapy in septic/vasoplegic shock is an improvement in hemodynamic stability, accompanied by a reduction in vasopressor requirements, as summarized in a recent review article [34]. In total, 33 eligible articles, including 353 patients, were analyzed, showing evidence of a significant reduction in norepinephrine (NE) requirements after treatment; median NE dose decreased from 0.55 to 0.09 μg/kg/min (*p* < 0.001). An analysis of four studies with control groups that included 140 patients in total revealed a large and significant pooled effect size, indicating a decrease in vasopressor requirements at 24 h (with a standardized mean difference of 1.64[95% CI: 0.53–2.76]), though data were characterized by high heterogeneity (I^2^ = 85.09%) [35,36,37,38]. Despite these promising results, several questions—including appropriate patient selection, timing of initiation, and dosing of CytoSorb^®^ therapy—remain unanswered.

### 3.4. Patient Selection

The decision process to use CytoSorb^®^ should always begin with the identification of the right patient candidates who are most likely to benefit from the therapy.

In one of the earliest studies, Friesecke et al. prospectively studied cytokine adsorption in 20 patients with refractory shock. This was defined as an already elevated (>0.3 µg/kg/min) and further increasing vasopressor dose (over the preceding 2 h) required to maintain a mean arterial blood pressure above 65 mmHg or already high lactate (>2.9 mmol/L), and further increasing levels, despite standard early goal-directed shock therapy, for at least six hours [39]. Following the initiation of CytoSorb^®^, the norepinephrine dose was significantly reduced after 6 (*p* = 0.03) and 12 h (*p* = 0.001), while lactate clearance also showed a significant improvement. Shock reversal was achieved in 13 (65%) patients; 28-day survival was 45% compared to a predicted mortality from the sequential organ failure assessment (SOFA) score of >80%.

Contrasting results emerged from the first clinical trial ever conducted with CytoSorb^®^ (2008–2011) [40]. In this randomized, controlled, open-label, multicenter trial, Schaedler et al. reported on the use of CytoSorb^®^ for 6 h daily for 7 days versus standard of care in 97 mechanically ventilated patients with a confirmed diagnosis of sepsis or septic shock, as well as acute lung injury or acute respiratory distress syndrome (ARDS), the latter established within the preceding 72 h and confirmed by clinical, radiological, or physiologic findings [40]. Safety was demonstrated in the technical domain (no interruption of therapy was necessary due to technical problems, and no clotting), as well as clinically (no significant impact on albumin and platelets; mortality unaffected after adjustment for higher baseline disease severity in the CytoSorb^®^ patients). Efficacy was demonstrated by the proof of significant removal of IL-6 in the measurements pre- and post-adsorber, although no significant effect on systemic blood levels was observed. Importantly, patients with refractory septic shock were excluded from participation. Moreover, many patients did not have severe hyperinflammation at study inclusion (median IL-6 565 pg/mL), which is below the levels where, according to current knowledge, CytoSorb^®^ exhibits its full cytokine removal effect due to the concentration-dependent nature of substance removal.

In a retrospective propensity score matching analysis, Scharf et al. included 19 matched patient pairs with an IL-6 > 10,000 pg/mL [41]. Whilst they found no difference in IL-6 reduction, hemodynamic stabilization, or mortality between patients receiving CytoSorb^®^ treatment and the matched patient cohort, it is noteworthy to mention that patients in the CytoSorb^®^ group were evidently more ill, as indicated by significantly higher IL-6 levels, simplified acute physiology score (SAPS) II, requirement for continuous renal replacement therapy (CRRT), norepinephrine doses, and lactate levels. However, this study primarily analyzed the effects of the first adsorber used on each patient, where the required minimum duration for CytoSorb^®^ use was merely 90 min, which is a duration that is arguably insufficient to significantly lower IL-6 levels exceeding 10,000 pg/mL.

A recent study by Kogelmann et al. showed that a dynamic scoring system (DSS), devised by the researchers, might be instrumental in pinpointing patients who could potentially benefit from hemoadsorption [42]. This system evaluates and assigns scores based on lactate concentrations (and their fluctuations over a span of 6 h), norepinephrine doses (and their change over 6 h), the necessity for a second catecholamine/vasopressor, use of hydrocortisone, and fluid boluses. These score parameters are duly recorded at the time of diagnosis (T0) and then again 6 h post-diagnosis (T6). A cumulative score exceeding six points, indicating a patient’s deteriorating condition despite 6 h of standardized treatment, signifies a refractory shock scenario. In such cases, the incorporation of hemoadsorption as an adjunctive therapeutic measure becomes pertinent. The study’s findings highlighted that the escalated dynamic scoring system (DSS) scores correlated with a surge in mortality rates. Furthermore, prolonged delays preceding the commencement of CytoSorb^®^ therapy (subsequent to a septic shock diagnosis) correlated with increased mortality rates. Specifically, CRRT patients diagnosed with septic shock, with a CytoScore above eight, seemed to derive significant benefits from the early instigation of CytoSorb^®^ therapy within 12 h post-diagnosis. This was in contrast to the control CRRT patients not treated with CytoSorb^®^, as evident from the marked disparity in mortality rates.

Two recent propensity-matched studies, used to address baseline differences between the groups, also support the concept of focusing specifically on patients who were not responding rapidly to the standard of care (SOC) and had high levels of NE needs and lactate [35,43]. Rugg et al. found, by genetic matching, that both in-hospital- and 28-day-mortality were significantly lower in the CytoSorb^®^ group as compared to the controls. In alignment with these findings, Brouwer et al. observed a 27% reduced mortality in 67 CytoSorb^®^ and continuous veno-venous hemofiltration (CVVH) treated patients when applying stabilized inverse probability of treatment weights (sIPTW), propensity matching in comparison to 47 CVVH patients being treated with CVVH alone [43]. In a follow-up study, the authors also demonstrated a long-term benefit in terms of survival for patients having undergone CytoSorb^®^ treatment [44]. They were also able to demonstrate in this study that the survival benefit was found in patients having lactate below 7 mM/L, similar to that found by the independent study of Rugg et al. [35].

These results underscore the potential importance of commencing CytoSorb^®^ therapy early, particularly before lactate exceeds 6.5 mmol/L.

Although not strictly related to the topic of sepsis and septic shock, the quality of patient selection may also have had a pronounced impact on the varying outcomes of recent infective endocarditis (IE) studies. One randomized control trial (RCT) did not provide specific inclusion criteria, only specifying the presence of infective endocarditis requiring surgery [45], resulting in non-significant results between the hemoadsorption group versus controls. Conversely, in a propensity-matched study on IE patients requiring surgery, only those with a EuroSCORE II ≥ 8% were included [46]. Their results suggested that CytoSorb^®^ seemed to diminish the severity of postoperative sepsis, reduced sepsis-associated mortality, and was associated with a significantly lower rate of postoperative respiratory failure requiring reintubation. Consistent with these findings, a retrospective study of prospectively collected data, encompassing consecutive high-risk patients (EuroSCORE II 12%) undergoing surgery for confirmed staphylococcus aureus infective endocarditis, showed that sepsis-related mortality, as well as 30-day and 90-day overall mortality, were also significantly reduced in the hemoadsorption group when compared to the controls [47]. This reiterates that meticulous patient selection, as opposed to a universal approach, is pivotal to maximize the clinical benefits of CytoSorb^®^ therapy and improve patient outcomes.

So, taken together, CytoSorb^®^ therapy should be considered in refractory septic or vasoplegic shock patients not responding to standard therapy who also exhibit clear signs of systemic hyperinflammation. A recently introduced dynamic scoring system (DSS) may further aid decision making since patients with a score ≥ 8 and early initiation of CytoSorb^®^ had significantly improved outcomes compared with renal replacement therapy alone, termed “control” patients. Classical biomarkers like IL-6, procalcitonin (PCT), or ferritin can further support decision making, but are not considered a mandatory prerequisite given a classical clinical picture as outlined above is present.

Despite the increased likelihood of therapeutic success in cases with clearly elevated levels of soluble mediators, such as IL-6 and PCT, which can potentially be adsorbed, treatment should not be withheld in centers with limited access to these biomarkers when clinical indicators strongly suggest the presence of refractory, hyperinflammatory vasoplegic shock. From a feasibility standpoint, clinical signs should remain the primary guideline for initiating treatment in such settings.

### 3.5. Timing

The timing of therapy initiation is the second critical component for clinicians to consider when deciding on CytoSorb^®^ therapy.

Several studies have reported on the timepoint of initiation of CytoSorb^®^ therapy, however, with quite some heterogeneity regarding the reference events (e.g., after intensive care unit—ICU admission, after diagnosis of septic shock, after the start of standard therapy, etc.).

In their proof-of-concept randomized controlled pilot study, Hawchar et al. [37] investigated the effects of early extracorporeal cytokine removal with CytoSorb applied as a standalone treatment in 10 vs. 10 patients with early (<24 h) septic shock. Patients with the following criteria were included: mechanical ventilation; norepinephrine > 10 μg/min; PCT > 3 ng/mL; and without the need for renal replacement therapy (RRT). In the CytoSorb^®^ group, norepinephrine requirements, as well as PCT concentrations, decreased significantly compared to the controls.

In the study by Friesecke et al., CytoSorb^®^ treatment was started after a median of 7.4 h post-ICU admission, yielding promising outcomes as already stated in the ‘Patient Selection’ section [39].

In the retrospective, the propensity-matched, single-center study by Rugg et al., septic shock patients receiving CytoSorb^®^, in addition to RRT (*n* = 42), were analyzed and compared to the closely matched control patients (*n* = 42) [35]. In this cohort, CytoSorb^®^ was started, on average, 21.4 h after ICU admission. The catecholamine requirements remained unchanged in the control patients, but in those treated with CytoSorb^®^, the levels were reduced by half to 0.26 µg/kg/min within 24 h of therapy initiation. Furthermore, in-hospital, as well as 28-day mortality, were significantly lower in the CytoSorb^®^ group (35.7% vs. 61.9%, *p* = 0.015 and 21.4% vs. 47.6%, *p* = 0.029, respectively).

In the above-mentioned Schaedler et al. RCT, no specific criteria were defined as thresholds when CytoSorb^®^ therapy should be initiated [40]. However, patients had to have at least 24 h of antibiotic therapy before initiation of hemoadsorption. So, in contrast to Rugg, CytoSorb^®^ was started more than 24 h after the start of standard therapy, which—based on our current understanding of ideal timing—may be a little too late.

A recent study by Wendel Garcia et al. suggested that early/late timing might not only be defined by hours after a reference event, such as the start of standard therapy, but also by the clinical status of the corresponding patient [48]. This retrospective, investigator-initiated observational study described the use of CytoSorb^®^ in 48 patients vs. 48 historical controls in refractory septic shock. CytoSorb^®^ patients had a SOFA score of 14, a norepinephrine requirement of 0.7 µg/kg/min, and a serum lactate of 5.8 mmol/L at baseline, indicating quite an advanced disease state. The rather negative results—CytoSorb^®^ therapy was not associated with reductions in IL-6 levels or vasopressor requirements, and in fact showed an increased hazard of death—are in contrast to other observational studies with a similar setup [35,43] and further support the need for properly designed prospective trials in the field.

Recently, Kogelmann et al. developed the previously mentioned DSS to assess patients in early refractory septic shock and to support decision making when commencing CytoSorb^®^ therapy [42]. A significant 56-day ICU and hospital survival advantage in CytoSorb^®^ patients was seen when therapy was started early (<12 h after diagnosis of septic shock), despite patients having higher lactate levels and norepinephrine needs compared to the two other investigated groups with a longer therapy delay (12–24 h and >24 h). Advanced statistical analysis showed that each additional hour in delaying CytoSorb^®^ therapy further increased the odds of mortality at day 56 by 1.5%, which was also significant (*p* = 0.034).

Based on the above, CytoSorb^®^ therapy should ideally be started early and within the first 12 h after diagnosis of septic or vasoplegic shock. In general, use should occur before irreversible organ damage has occurred, and lactate values > 6.5 mmol/L may serve as predictors of a worse outcome.

### 3.6. Dosing

Duration (overall length of treatment), as well as intensity (time interval between adsorber changes), represent very important “dosing” variables for the optimization of CytoSorb^®^ therapy. According to the “Instructions for Use” (IFU) of the device, the maximum treatment time per adsorber is 24 h, while in everyday clinical practice, many institutions have discovered that more frequent exchanges, especially at the start (i.e., every 12 h), are critical for achieving the desired effect. Since shock reversal should be accomplished as soon as possible, it is important to achieve the maximal therapeutic effect quickly by starting with a higher dose (similar to the “bolus”, or “loading dose” concept with drug therapy). After shorter exchange intervals of 8–12 h on the first and possibly also the second treatment days, or once stabilization has been achieved then less frequent (i.e., every 24 h) adsorber changes may be sufficient (i.e., “maintenance dose”). This approach, which intends to ensure the rapid onset and continuous maintenance of high removal rates during device use, is being prospectively evaluated in the ongoing PROCYSS randomized control trial (ClinicalTrials.gov Identifier: NCT04963920). On the other hand, there is also limited information on the question of how long treatment should be maintained for, or conversely, when the best time to stop treatment is, both from a clinical but also economic point of view.

The study by Schaedler et al. [40] did not find a therapeutic benefit, but the duration of treatment was 6 h only with an 18 h break before the subsequent session, and disease severity was also substantially lower compared to other studies [39,48]. In the study by Scharf et al., although patients had IL-6 levels higher than 10,000 pg/mL, the median treatment duration was only 9 h (range: 7–12 h) with no change of the adsorber during the study period, which may have resulted in the observed lack of effect [41].

Generally, sepsis/septic shock patients are likely to require both a more intense and longer treatment to show sufficient and sustained therapeutic success. Nevertheless, even when applied for only 24 h (mostly only one adsorber used in total) in a cohort of patients with septic shock with [35] or without [37], the need for renal replacement therapy, CytoSorb, use was still associated with improved hemodynamic status [35,37] and improved survival [35].

Friesecke et al. [39] applied a mean of 3.0 ± 1.5 adsorbers per patient, which were changed every 8–12 h, depending on the patient’s clinical response, and treatment was discontinued if no further effect on IL-6 was to be expected. As mentioned earlier, with that treatment regimen, norepinephrine requirements and lactate clearance improved significantly with a 28-day survival of 45% compared to the predicted mortality from the SOFA score of >80%.

An intriguing approach was proposed by Schultz et al. recently [49]. The authors suggest dosing of CytoSorb^®^ therapy via the amount of blood purified (ABP) in L/kg. ABP is calculated as the duration of CytoSorb^®^ treatment (in minutes), times the blood flow through CytoSorb^®^ (mL/min), divided by the actual body weight (kg) (plus a correction factor of 1/1000 to result in L/kg). However, this formula does not address the impact of adsorber changes, which were conducted around every 26 h on average. They found that the clinical effects of CytoSorb^®^ were related to the amount of blood purified and identified the necessary threshold for ABP as at least 13 L/kg. The authors concluded: “*These results suggest that hemoadsorption with CytoSorb^®^ might contribute to better survival in septic shock and severe CRS (cytokine release syndrome), provided that the applied dose is high enough” [49]*.

The largest dataset available to date is the CytoSorb^®^ international registry, which includes 1437 patients treated with CytoSorb^®^ [50]. In the sepsis cohort (*n* = 939), the median number of adsorbers used was two, and treatment lasted for a median of 43 h. At the end of the course of hemoadsorption, 85% of the patients were alive, and physicians reported an improvement based on their overall subjective impression in 54% of cases. Although there was no significant improvement in the overall SOFA score, the cardiovascular and respiratory SOFA sub-scores improved significantly after therapy (median change of −0.4 and −0.2 score points, respectively) as compared to baseline.

In summary, CytoSorb^®^ therapy should consistently be maintained (with the replacement of adsorbers as needed) until adequate hemodynamic stabilization (e.g., NE dose permanently ≤ 0.05 µg/kg/min) is achieved. To ensure maximum removal capacity, adsorber exchanges for the first two days of treatment should be considered after every 8–12 h, especially in cases of persistent hemodynamic instability. In general, each adsorber must be changed at the latest when the maximum therapy duration of 24 h is reached, and the treatment is to be continued.

### 3.7. Therapeutic Goals

The therapeutic strategy of CytoSorb^®^ hemoperfusion centers around modulating the host response to infection and mitigating organ dysfunction at an early point in time. To verify this objectively and reproducibly, proximal clinical endpoints, like change in (∆) SOFA [51], cumulative vasopressor dose [52], lactate clearance [53], time spent on mechanical ventilation, days on renal replacement therapy, and, perhaps, length of ICU stay, should be taken into account.

Of far greater importance to the patient, however, are more distal endpoints such as 28- and 90-day mortality, the improvement of which are, of course, the ultimate goals of therapy. However, it is also clear that only RCTs with a large number of patients can demonstrate a survival benefit from hemadsorption therapy.

### 3.8. Safety

As the device removes not only cytokines but various other substances as well, there is the theoretical concern that the potential unwanted removal of otherwise important molecules (e.g., certain drugs) may be disadvantageous and potentially impact the safety profile.

The risk of the unintended removal of concomitantly administered drugs in critically ill patients is an important issue that needs consideration with the use of all extracorporeal therapies. The clinical relevance of potential drug removal by CytoSorb^®^ depends not only on the impact of the device, but also on drug-specific variables such as volume of distribution, protein binding, and elimination half-life. The duration of device exposure, and initiation of drug administration versus steady-state conditions, are further aspects requiring consideration when assessing the clinical relevance of potential drug removal. CytoSorb^®^ drug adsorption kinetics show that most of the adsorption occurs in the first few hours of device exposure. Therefore, for drugs prone to adsorption, an increased loading dose and/or an additional dose after the first 1–2 h of treatment should be considered. A recent systematic review on drug removal by CytoSorb^®^ provides more detail on the topic and also some guidance for clinical decision making [54].

### 3.9. Procedural Details

While detailed procedural steps are beyond the scope of this manuscript and can be found in the instructions for use (IFU), we will briefly outline the general procedure and precautions required to apply the device effectively and safely. CytoSorb^®^ is intended for use in conjunction with standard commercially available bloodlines compatible with the utilized pump system. The cartridge can be integrated into all standard extracorporeal blood pumps, including intermittent hemodialysis, continuous renal replacement therapy (CRRT), extracorporeal membrane oxygenation (ECMO), and cardiopulmonary bypass (CPB). Pressure monitoring of the bloodline between the blood pump and CytoSorb^®^ is recommended. When used with ECMO, CytoSorb^®^ should be placed in a shunt off the primary flow, flow monitoring should be used with the flow rate adjusted to ensure delivery of the desired flow to the patient (≤700 mL/min). Of note, a flow rate of 600 mL/min through the adsorber circuit will shunt approximately 20% of the blood flow from the patient. In combination with renal replacement therapy, the device can be incorporated either upstream (proximal) or downstream (distal) of the hemofiltration/dialysis device. Moreover, the priming process is crucial, with the blood lines requiring thorough priming with saline to prevent air entry, which could lead to clotting and reduced device efficacy. Multiple priming options, including gravity and pump priming, are available. During treatment, diligent monitoring of pressure in the extracorporeal circuit is essential, as is a visual inspection of the CytoSorb^®^ cartridge for clotting or blood leaks. Obstructions, fitting security, and air within the circuit should also be periodically assessed. Upon treatment completion, it is imperative to follow the provided IFU for the bloodlines and blood pump circuit, returning the blood in the device and lines to the patient as standard practice. CytoSorb^®^ and the bloodlines are single-use devices and should be discarded in an appropriate biohazard waste receptacle. Reuse attempts could result in secondary infection, device clotting, or biohazardous situations, and, therefore, should be avoided.

### 3.10. Anti-Infectives

Schneider et al. also provided data on the removal of various anti-infective drugs by CytoSorb^®^, compared to sham hemoperfusion, in a highly standardized and controlled pig model. The tested drugs were beta-lactams (for example: classical antibiotics such as, e.g., meropenem or piperacillin), antifungals (such as fluconazole), aminoglycosides (tobramycin), and other types of anti-infectives (such as linezolid or clindamycin). Based on the findings, hemoadsorption with CytoSorb^®^ appears to have a limited effect on the pharmacokinetics of the majority of drugs tested. However, clearance of fluconazole, linezolid, and liposomal amphotericin B appeared to be increased by the procedure. Nevertheless, the authors state that any required dose modification would likely be minor [55].

Recent investigations of CytoSorb^®^’s impact on antibiotic levels in critically ill patients are in line with these findings. Liebchen et al. confirmed in a study of 25 critically ill patients that no dose adaptations for meropenem are required [56], whereas, for linezolid, it has been shown that an additional loading dose of 600 mg after the start of CytoSorb^®^ therapy might help to reduce the risk of subtherapeutic levels [57].

### 3.11. Anticoagulation

The need and type of anticoagulation with CytoSorb^®^ therapy are similar to standard strategies used with many other extracorporeal circuits during clinical practice. Options for anticoagulation include unfractionated heparin, citrate, or other anticoagulant strategies, depending on individual patient factors, coagulation profiles, and institutional protocols. Systemic heparinization is typically employed, with an aPTT of 60–80 s or an ACT of 160–210 s serving as adequate parameters for CytoSorb^®^ therapy. Regular monitoring of aPTT or ACT is essential to maintain appropriate anticoagulation levels. Alternatively, regional anticoagulation with citrate can be considered, involving the initial dose, blood flow rate, and meticulous control and adjustment of calcium and citrate according to the designated protocol. Monitoring ionized calcium levels, both within the CRRT circuit and the patient, is advised at the initiation of treatment and at regular intervals. The determination of the appropriate dosage and target values is ultimately at the discretion of the attending physician. It is important to note that usage of CytoSorb^®^ in hemoperfusion (stand-alone) mode without a hemofilter, heparin anticoagulation, is the preferred approach. Careful consideration of the appropriate anticoagulation strategy is essential to maintain optimal circuit function, prevent clotting, and to ensure the safe and effective application of CytoSorb^®^ therapy. In conclusion, the safety profile of CytoSorb^®^ therapy appears favorable based on existing data. However, efforts are ongoing to further expand the database through retrospective and prospective data collection. Notably, the prospective, international COSMOS-registry (ClinicalTrials.gov Identifier: NCT05146336), is currently underway to evaluate the performance of CytoSorb^®^ in a variety of critical care applications. COSMOS is collecting high fidelity, real-world data, to evaluate the utilization patterns and clinical performance of CytoSorb^®^ therapy and will undoubtedly provide invaluable insights on patient selection, timing, and dosing of therapy, as well as the further evaluation of safety in real-world practice.

## 4. Conclusions

While there exists a substantial body of literature, ongoing research aims to address still outstanding important questions, such as the optimal selection of patient groups most likely to benefit, the ideal timing for CytoSorb^®^ initiation, the frequency of adsorber exchanges, therapy duration, and investigation into potential unintended removal of concomitant medications [58]. This review summarizes the insights gained from the clinical use of the device in patients with septic vasoplegic shock over the past decade (Table 1, for the available literature cf. Supplementary Table S1). Based on the presently available data, an expert consensus has been reached on this practical guidance for the very important application of the technology in septic vasoplegic shock. With more than 200,000 treatments administered worldwide and supported by a growing body of evidence, CytoSorb^®^ hemoadsorption represents a promising and safe adjuvant treatment option for critically ill patients in severe hyperinflammatory conditions that do not respond to standard medical therapy.

## Figures and Tables

**Table 1:** Best practice suggestions for the use of CytoSorb in septic/vasoplegic shock.

**Patient Selection**	Consider in refractory septic/vasoplegic shock unresponsive to SOC (CytoScore > 6) [42]. Patients should have obvious signs of ongoing hyperinflammation. If available, soluble markers of inflammation should be clearly elevated (e.g., Il-6, PCT, ferritin).
**Timing**	Start within 12 but not later than 24 h after diagnosis of septic/vasoplegic shock.
**Dosing**	Change adsorber every 8–12 h during the first day or two of therapy. Later, change the adsorber every 24 h. Maintain therapy until hemodynamic stabilization (e.g., NE dose < 0.05 µg/kg/min) is reached.
**Concomitant Medication**	For drugs prone to adsorption (including anti-infectives), consider increased loading doses and/or additional doses after 1–2 h after initiation of CytoSorb therapy [54,55]. Therapeutic drug monitoring (TDM) at regular intervals is recommended if available.

SOC, standard of care. Il-6, interleukin 6. PCT, procalcitonin. NE, norepinephrine.

## Data Availability

Data are available from the corresponding author upon reasonable request.

## Supplementary Materials

The following supporting information can be downloaded at: https://www.mdpi.com/article/10.3390/jcm12237199/s1, Table S1: Outcomes, safety, timing, and dosing in published clinical studies using CytoSorb(R).
