# Supplementary material for: Adjunctive Hemoadsorption Therapy with CytoSorb in Patients with Septic/Vasoplegic Shock: A Best Practice Consensus Statement
Source: J Clin Med. 2023 Nov 21;12(23):7199. doi: 10.3390/jcm12237199 (PMC10707447; doi:10.3390/jcm12237199)
Supplement: Supplementary file 1 [file jcm-12-07199-s001.zip › jcm-2657983-supplementary.pdf]

Supplementary Table S1. Outcomes, safety, timing, and dosing in published clinical studies using CytoSorb(R).

Reference Details	Number and Type of Patients and number (n=)	Outcomes	Additional Information (safety, timing, dosing, duration of use)
Poli et al. [27]	Elective cardiac surgery (n= 15 vs 15)	There was no statistically significant difference in factors V, VIII, IX, XI and XII nor in vWF activity between pre- and post-adsorber measurements. A small but statistically significant decrease in AT and FII was observed (resp. from 70.4 ± 15.3 to 66.6 ± 16.5, p = 0.006, and 61.6 ± 16.2 to 57.3 ± 16.3, p = 0.03).	CytoSorb during CPB was not associated with an increased incidence of adverse events. Intraoperative use of CytoSorb (mean bypass duration 145 (130-183) mins).
Jansen et al. [33]	Healthy volunteers (n=12 vs 12)	Compared to the control group, use of CytoSorb resulted in significantly lower plasma levels of TNF, − 58%, p < 0.0001, interleukin (IL)-6 (− 71%, p = 0.003), IL-8 (− 48%, p = 0.02) and IL-10 (− 26%, p = 0.03) during the first LPS challenge. No differences in cytokine responses were observed during the second LPS challenge on day 7 when CytoSorb was not used.	The absence of any device-related adverse events in both our study and also in other available published reports suggests that the safety profile of CytoSorb therapy is favorable. One CytoSorb adsorber used for 6 hours. Blood flow rate 250 mL/min.
Rugg et al. [35]	Septic shock (n= 42 vs 42)	While remaining fairly constant in the controls, the catecholamines decreased to 0.26 (0.11–0.40) µg/kg/min within 24 h after initiation of CytoSorb therapy. In-hospital mortality was significantly lower in the CytoSorb group (35.7% vs. 61.9%; p = 0.015).	CytoSorb was mounted a median 21.4 h after ICU admission. 38 / 42 CytoSorb patients received one single treatment, n = 3 received two and one single patient received a total of six CytoSorb treatments. Standard duration of CytoSorb treatment was 24 hours.
Mehta et al. [36]	Major Aortic Surgery (n= 8 v 8)	IL-6 levels were significantly lower in the CytoSorb group compared with the control group at all time points after the intervention. A significantly reduced requirement of norepinephrine was seen in the CytoSorb group at the T2 time point (0.019 v 0.048 µg/kg/min; p = 0.043). The PF ratio was better in the CytoSorb group, and duration of postoperative ventilation was also shorter. ICU stay (2.28 d v 3.0 d; p = 0.037) and hospital stay (4.71 d v 5.71 d; p = 0.007) was shorter in the CytoSorb group.	CBP time 174 (48.4) mins. Intraoperative use of CytoSorb.

Hawchar et al. [37]	Septic shock (n= 10 vs 10)	Overall SOFA scores did not differ between the groups. In the CytoSorb-group norepinephrine requirements and PCT concentration decreased significantly (norepinephrine: CytoSorb: T0 = 0.54[IQR:0.20–1.22], T48=0.16[IQR:0.07–0.48], p=.016; Controls: T0=0.43[IQR:0.19–0.64], T48=0.25[IQR:0.08–0.65] μg/kg/min; PCT: CytoSorb: T0 median= 20.6[IQR: 6.5–144.5], T48=5.6[1.9–54.4], p = .004; Control: T0 = 13.2[7.6–47.8], T48 = 9.2[3.8–44.2]ng/mL). Big-endothelin-1 concentrations were also significantly lower in the CytoSorb group (CytoSorb: T0 = 1.3 ± 0.6, *T24 = 1.0 ± 0.4, T48= 1.4±0.8, *p = .003; Control: T0 = 1.1 ± 0.7, T24=1.1 ± 0.6, T48= 1.2 ± 0.6 pmol/L, p = .115).	There were no CytoSorb therapy-related adverse events. One adsorber was used for 24 hours. Blood flow rate 250 – 400 mL/min.
Akil et al. [38]	Pneumonic sepsis requiring ECMO (n= 13 vs 7)	All patients survived in the CytoSorb group, where the 30-day mortality rate reached 57% in the control group. After CytoSorb therapy there was an instantly observed significant reduction in PCT and CRP levels compared with the control group. Within 48 hours, the initial high doses of catecholamine could be weaned off only in the CytoSorb group.	Non any adverse events related to the treatment and the adsorber were observed. Average 3 adsorbers per patient for up to three days. Blood flow rates kept between 200 and 400 mL/min.
Friesecke et al. [39]	Refractory septic shock (n=20)	CytoSorb" treatment was started after 7.8 ± 3.7 h of shock therapy. Following the initiation of adsorption therapy, noradrenaline dose could be significantly reduced after 6 (-0.4 lg/kg/min; p = 0.03) and 12 h (-0.6 lg/kg/min; p = 0.001). Lactate clearance improved significantly. SOFA-scores on day 0, 1 and 2 remained unchanged. Shock reversal was achieved in 13 (65%) patients; 28-day survival was 45%.	No serious adverse effects of the therapy were observed. Mean of three adsorbers used per patient which were exchanged every 8 – 12 hours per treatment.
Schaedler et al. [40]	Sepsis or septic shock (n= 47 vs 50)	Significant removal of IL-6 in the measurements pre- and post-adsorber, although no significant effect on systemic blood levels was observed. Mortality unaffected after adjustment for higher	No interruption of therapy was necessary due to technical problems, and no clotting, as well as clinically (no significant impact on albumin and platelets. Seven adsorbers used for 6 hours per day (7 days). Blood flow rates 200 – 250 mL/min.

		baseline disease severity in the CytoSorb patients	
Scharf et al. [41]	Cytokine storm (IL6 >10,000 pg/ml, n= 19 vs 19)	No difference in IL-6 reduction, hemodynamic stabilization, or mortality between patients receiving CytoSorb treatment and the matched patient cohort, however, patients in the CytoSorb group were more ill, as indicated by significantly higher IL-6 levels, SAPS II score, requirement for CRRT, norepinephrine doses, and lactate levels.	Minimum duration of use 90 mins. Only one treatment cycle used for analysis.
Kogelmann et al. [42]	Refractory septic shock (n= 198 v 304)	Developed dynamic scoring system to allow assessment of clinical development of patients in early septic shock. Use of same was able to detect populations with distinct mortality patterns. Early start of CytoSorb associated with significantly improved survival.	Average three adsorbers used per patient.
Brouwer et al. [43]	Septic shock (n= 67 vs 49)	27 % reduced mortality in CytoSorb vs CVVH only patients when applying stabilized sIPTW propensity matching. Independent predictors of 28-day mortality in the CytoSorb group were the presence of pneumosepsis, higher levels of lactate at the start of CytoSorb and older age.	Average two adsorbers used per patient, exchanged every 24 hours. Filtration rate 250 – 400 mL/min.
Brouwer et al. [44]	Septic shock (n= 67 vs 49) Follow up study from 2019 study.	Independent factors associated with long-term outcome in CytoSorb patients were baseline lactate levels, age in the presence of comorbidity and presence of abdominal sepsis A lactate level above 6.0 mmol/L at the start of CytoSorb therapy had a positive predictive value of 79% for mortality (p = 0.013).	As above (average two adsorbers used per patient, exchanged every 24 hours. Filtration rate 250 – 400 mL/min.)
Diab et al. [45]	Infective endocarditis requiring surgery (n= 138 vs 144)	Levels of IL-1β and IL-18 at the end of CPB were significantly lower in the CytoSorb than controls. The primary outcome, ΔSOFA, did not differ between the CytoSorb and controls.<br>Mortality at 30 days, duration of mechanical ventilation, and vasopressor and renal replacement therapy did not differ between groups.	No significant differences in adverse events between groups. Intraoperative use of CytoSorb. Total duration of adsorber use 2.31 ±1.45 hours.

Haidari et al. [46]	High risk infective endocarditis requiring surgery (n= 35 vs 35)	Postoperative sepsis occurred in 14 patients (4 death) in the CytoSorb group and 16 control patients (11 deaths), p = 0.041. In-hospital mortality was 34% in the CytoSorb group versus 43% in the controls. Cumulative vasopressor dose was lower and postoperative course of SOFA score normalized significantly faster in the CytoSorb group.	Intraoperative use of CytoSorb. CBP time 127 (105 – 177) mins.
Haidari et al. [47]	Confirmed staph aureus infective enddarditis requiring surgery (n=75 vs 55)	Significantly decreased vasoactive-inotropic score was observed in the CytoSorb group at all time points [6 h: 6.0 (0–17) vs 17 (3–47), p = 0.0014; 12 h: 2 (0–8.3) vs 5.9 (0–37), p = 0.0138; 24 h: 0 (0–5) vs 4.9 (0–23), p = 0.0064; 48 h: 0 (0–2.1) vs 0.1 (0–13), p = 0.0192; 72 h: 0 (0) vs 0 (0–5), p = 0.0014]. Importantly, sepsis-related mortality (8.0% vs 22.8%, p = 0.02) and 30-day (17.3% vs 32.7%, p = 0.03) and 90-day overall mortality (21.3% vs 40%, p = 0.03) were also significantly lower with hemoadsorption.	CytoSorb was safe and easy to use. Intraoperative use of CytoSorb. CPB time 133.2 ± 72.8.
Wendel Garcia et al. [48]	Refractory septic shock (n= 48 vs 48)	CytoSorb therapy was not associated with reductions in IL-6 levels or vasopressor requirements.	CytoSorb started within 24 hours of shock onset, and provided for three x 24 hr sessions.
Schultz et al. [49]	Septic shock (n= 70)	The amount of blood purified was higher in survivors than in non-survivors (8.5 ± 4.4 vs. 6.1 ± 3.6 l/kgBW, p = 0.017). Three clusters (<6 l/kgBW, 6-13 l/kgBW and ≥ 13 l/kgBW) identified with a linear dose response relation between blood purification volume and survival, which was best in the highest volume cluster (83.3%; p = 0.045)	Three CytoSorb adsorbers used on average for 85 ± 53.8 hrs. Amount of Blood Purified (ABP) in l/kg. ABP calculated as the duration of CytoSorb® treatment (in minutes) times the blood flow through CytoSorb® (ml/min) divided by the actual body weight (kg) (plus a correction factor of 1/1000 to result in l/kg).
Hawchar et al. [50]	Registry (n=1437 including 939 in sepsis cohort.)	There was no significant difference in the primary outcome of mortality, but there were improvements in cardiovascular and pulmonary SOFA scores and a reduction in PCT, CRP and IL-6 levels. Evaluation of the overall effect: minimal improvement (22%), much improvement (22%)	Analysis of the Registry data suggest that CytoSorb therapy is safe. Median number of CytoSorb adsorbers 2, with treatment lasting 43 hours.

		and very much improvement (10%), no change observed (30%) and deterioration (4%).	

Legend; Confidence Interval (CI), Creatinine Kinase (CK); Intensive Care Unit (ICU); Cardiopulmonary Bypass (CPB); Tumur Necrosis Factor (TNF); Interleukin (IL); Lipopolysaccharides (LPS); Sequential Organ Failure Assessment (SOFA); Procalcitonin (PCT); Extracorporeal Membrane Oxygenation (ECMO); C-Reactive Protein (CRP); Simplified Acute Physiology Score (SAPS) II; Continuous Renal Replacement Therapy (CRRT); Continuous Veno-Venous Hemofiltration (CVVH); Stabilized Inverse Probability of Treatment Weights (sIPTW);
